# Design of Functional Pluronic-Based Precursors for Tailoring Hydrogel Thermoresponsiveness and Cell-Adhesive Properties

**DOI:** 10.3390/ma16072749

**Published:** 2023-03-29

**Authors:** Giulia Camana, Mirko Tavano, Min Li, Franca Castiglione, Filippo Rossi, Francesco Cellesi

**Affiliations:** 1 Dipartimento di Chimica, Materiali ed Ingegneria Chimica “G. Natta”, Politecnico di Milano, Via Mancinelli 7, 20131 Milan, Italy; 2 Renal Research Laboratory, Fondazione IRCCS Ca’ Granda Ospedale Maggiore Policlinico, Via Pace 9, 20122 Milan, Italy

**Keywords:** Pluronic, F127, gel, Michael-type addition, thermoresponsive, cell adhesion

## Abstract

In this study, functional Pluronic F127 precursors were designed and synthesized for the preparation of thermosensitive hydrogels. Using linear Pluronic thioacetate and Pluronic multi-acrylate precursors, F127-based hydrogels were prepared through thioacetate deprotection-mediated Michael-type addition. The properties of these gels were compared to those obtained through free radical crosslinking of F127 diacrylate. Temperature was found to have a clear influence on gel swelling as a result of F127 thermoresponsiveness. The macromolecular architecture and functionality of the precursors were also optimized and characterized in terms of gelation kinetics and drug diffusion. In vitro tests were conducted on fibroblasts and endothelial cells to assess their response to cellular adhesion with Pluronic gels that were functionalized with an RGD peptide or pretreated with serum proteins to promote cell adhesion. This study provides a method for creating tailored hydrogels suitable for various biomedical applications, such as soft-tissue engineering, cell encapsulation, wound healing, and sustained delivery of therapeutic molecules.

## 1. Introduction

Poly(ethylene oxide)-poly(propylene oxide)-poly(ethylene oxide) (PEO-PPO-PEO) triblock copolymers, known as poloxamers or Pluronics, are thermoresponsive amphiphilic macromolecules used to develop in situ gel systems in aqueous solutions [1,2,3,4,5]. They are widely utilized in various pharmaceutical dosage forms, including oral, parenteral, ocular, topical, vaginal, and rectal, as they are FDA-approved, non-toxic, and non-irritant excipients [1,6]. Pluronic F127 (Poloxamer 407) with an average molecular weight of 12,600 Da and a PEO/PPO weight ratio of 70:30 [7] is one of the most widely used poloxamers in pharmaceutical preparations. Its high drug-solubilizing capacity, sustained-release properties, low toxicity, and compatibility with various biomolecules and chemical excipients render it a preferred choice among Pluronics of different molecular weights and PEO/PPO ratios [1,6,8]. Pluronic F127-based hydrogels have a unique thermosensitive property and undergo injectable sol–gel transition under physiological temperature [9], making them useful in various biomedical applications [10], such as soft-tissue engineering, cell encapsulation [1,7,11], adhesives for wound healing and surgery [12,13], bioink [14,15], and biomaterials for the sustained delivery of therapeutic molecules [8,16]. However, these hydrogels have typical characteristics of physically crosslinked materials, such as relatively poor mechanical properties and spontaneous dissolution in physiological fluids [1,7], which may limit their clinical applications despite their advantages. Previous studies proposed a gelation process based on both physical and chemical mechanisms, known as the “tandem process”, in which a polymeric liquid precursor derived from Pluronic is first thermally gelled and then covalently crosslinked by reacting polymer end groups [7,11]. This second-stage chemical gelation is achieved through a cell-friendly Michael-type addition of thiols onto acrylates, which can be obtained at physiological temperature and pH. The fast thermal gelation kinetics and harmless nature of the Michael-type addition enable the formation of stable crosslinked hydrogels suitable for cell encapsulation in various biomedical applications [7,11,17,18].

To obtain the “tandem” gels, Pluronic precursors with multiple functional sites necessary for crosslinking were designed. One approach involves using a Pluronic that contains two acrylate groups at its termini, which can crosslink in the presence of another Pluronic-based macromolecule functionalized with multi-thiol end groups [7]. However, a drawback of this approach is that free thiol groups may form disulfides by oxidation during storage or the crosslinking reaction [11,19]. This is due to the presence of oxygen in the atmosphere or dissolved in the gelling solution, which can result in unpaired groups still being present at the end of the process. To overcome this limitation, tetra-armed Pluronic analogous (Tetronic) end-functionalized with thioacetates and acrylates, respectively, can be used. Tetronics are poloxamines that present four PPO–PEO chains conjugated to an ethylene diamine central group, and they exhibit similar Pluronic physical gelation properties at physiological temperatures [11,20]. Thioacetates are used as protected Michael donors, which produce thiols via basic treatment only when the Michael-type addition is required. Moreover, the steric hindrance of the reacting groups of these star polymers does not significantly change during the course of the reaction [11].

On the other hand, the commercial availability of Tetronics with a similar PPO/PEO ratio as Pluronic F127, such as Tetronic T1107 for biological use, is relatively limited. Additionally, the presence of the ethylene diamine central group in Tetronic T1107 may have an impact on the final physicochemical properties of the resulting gel and its interactions with cells [21].

In this study, we present an alternative synthetic approach to functionalize Pluronic F127 using linear Pluronic thioacetate and Pluronic multi-acrylate precursors. This allows for the preparation of F127-based hydrogels through thioacetate deprotection-mediated Michael-type addition. The properties of these gels were compared to those obtained through free radical crosslinking of F127 diacrylate, which is a simpler polymeric precursor to synthesize. We optimized and characterized the macromolecular architecture and functionality of the precursors in terms of gelation kinetics, swelling, and diffusion. We also conducted in vitro tests on fibroblasts and endothelial cells to assess their response to cellular adhesion. To promote cell adhesion, the Pluronic gels were either functionalized with an Arginylglycylaspartic acid (RGD) peptide or pretreated with fetal bovine serum (FBS) proteins. In this way, these customized Pluronic-based hydrogels may be appropriate for different biomedical purposes, including soft-tissue engineering, cell encapsulation, wound healing, and sustained drug release.

## 2. Materials and Methods

Pluronic F127 (MW = 12,600, 70% PEG, composition confirmed with proton (^1^H) nuclear magnetic resonance (NMR) spectroscopy), allyl bromide, sodium hydride, 2,2′-Azobis(2-methylpropionitrile) (AIBN), thioacetic acid (purum, ≥ 95.0% (GC)), Tetronic 701 (MW = 3600, 10% PEG), acryloyl chloride (purity: 97.0%, contains <210 ppm MEHQ as stabilizer), triethylamine (purity ≥ 99.5%), tris(2-carboxyethyl)phosphine hydrochloride (TCEP) (purity 98%), sodium hydroxide (purity ≥ 97%, pellets), hydrochloric acid (37%), potassium phosphate monobasic, sodium phosphate dibasic, toluene, N,N-Dimethylformamide, ammonium persulfate (APS), tetramethylethyldiamine (TEMED), and ibuprofen (purity 98%) were purchased from Merck (Sigma-Aldrich, Milan, IT, USA) and used as received.

All the functionalized polymers obtained were characterized using a 400 MHz Bruker ^1^H NMR spectrometer and a Thermo Scientific Nicolet iS50 Fourier-transform infrared (FT-IR) spectrometer in Attenuated total reflection (ATR) mode. Dynamic light scattering (DLS) measurements were carried out with Malvern Zetasizer ZS (Malvern, UK). Size Exclusion Chromatography (SEC) analysis was performed with the Jasco GPC System in tetrahydrofuran (THF) at a flow rate of 1 mL/min at 35 °C, calibrated using polystyrene standards.

### 2.1. Synthesis of Pluronic F127 Allyl Ether (F127ALL)

A total of 10 g of Pluronic F127 (0.794 mmol, 1.59 meq of –OH groups) was dissolved in 150 mL of toluene under a nitrogen atmosphere and dried by refluxing through a Soxhlet extractor filled with 4 Å molecular sieves. After 3 h, the solution was cooled at room temperature and then immersed in an ice bath. The polymer was then deprotonated using 57 mg of NaH (1.5 eq per –OH group) and stirred for approximately 15 min. A total of 0.687 mL of allyl bromide (7.94 mmol, 5 eq per ONa group) dissolved in 10 mL of toluene was dropped into the solution using a dropping funnel, and the reaction mixture was stirred overnight at room temperature. The mixture was filtered using a Buchner filter, and the solvent was evaporated at the rotary evaporator. The resulting viscous oil was dissolved in 200 mL of dichloromethane and washed two times with 30 mL of deionized water saturated with sodium chloride. The solution was dried with sodium sulphate, filtered, the solvent evaporated, and then precipitated twice in cold diethyl ether. Conversion = 100% (from ^1^H-NMR data). Yield = 80%.

^1^H NMR (CDCl_3_): δ = 1.1 (m, 206H, PPG CH_3_ = 69 monomeric units), 3.4 (m, 68H, PPG CH = 68 monomeric units), 3.5 (m, 131H, PPG CH_2_ = 66 monomeric units), 3.65 (m, 924H, PEG chain protons = 231 monomeric units), 4.01−4.04 (dd, 4H, ‒CH_2_OCH_2_‒CH=CH_2_), 5.15–5.30 (m, 4H, ‒OCH_2_CH=CH_2_), 5.85–5.98 ppm (m, 2H, ‒OCH_2_CHCH_2_).

FT-IR (film on ATR plate): 2990–2790 (ν C-H), 1467 (δ_s_ CH_2_), 1342, 1279, 1242, 1097 (ν_as_ C-O-C), 962, 841 (ν_s_ C-O-C)cm^−1^.

### 2.2. Synthesis of Pluronic F127 Thioacetate (F127TA)

A total of 7 g of F127ALL (0.5 mmol, 1.11 meq of allyl groups) was dissolved in 150 mL of previously degassed toluene. After the addition of 0.91 g of AIBN (5.55 mmol; MW = 164.2; 5-fold excess compared to allyl groups), the solution was heated to 60–65 °C, and 0.13 mL of thioacetic acid was added repeatedly every 2 h (4 × 0.13 mL; 1.78 mmol, 1.6-fold excess per addition); then, the solution was stirred overnight at room temperature. Afterwards, the solvent was evaporated at the rotary evaporator, and the resulting viscous oil was dissolved in 200 mL of DCM and washed two times with 30 mL of deionized water saturated with sodium chloride. The solution was dried with sodium sulphate, filtered, the solvent evaporated, and then precipitated three times in cold diethyl ether. Conversion = 91% (from ^1^HNMR data). Yield= 87%.

^1^H NMR (CDCl_3_): δ = 1.1 (m, 206H, PPG CH_3_ = 69 monomeric units), 1.81–1.90 (broad q, 4H, ‒OCH_2_CH_2_CH_2_S‒), 2.32 (s, 6H, ‒SCOCH_3_), 2.92–2.97 (t, 4H, ‒CH_2_SCOCH_3_), 3.4 (m, 68H, PPG CH = 68 monomeric units), 3.49–3.52 (t, 4H, ‒OCH_2_CH_2_CH_2_S‒), 3.5 (m, 131H, PPG CH_2_ = 66 monomeric units), 3.65 ppm (m, 924H, PEG CH_2_ = 231 monomeric units).

FT-IR (film on ATR plate): 2990–2790 (ν C-H), 1692 (ν C=O), 1467 (δ_s_ CH_2_), 1342, 1279, 1242, 1097 (ν_as_ C-O-C), 962, 841 (ν_s_ C-O-C)cm^−1^.

### 2.3. Synthesis of Pluronic F127 Hexaacrylate (F127HA)

A total of 2 g of F127TA was dispersed in 10 mL of NaOH 0.2 M solution, and the mixture was left in an ice bath (2–3 °C) for 1.5 h under gentle stirring. The cold polymer solution was buffered with 7 mL of HCl 0.6 M solution and 1.5 mL of HCl 0.2 M solution to obtain a pH equal to 7.4. A total of 1.11 g of pentaerythritol tetraacrylate (315 mmol; equivalent tetraacrylate/thioacetate = 40) dissolved in 18.5 mL of DMF was then added to the polymer solution. Finally, 90 mg of TCEP (0.315 mmol; 1 equivalent per equivalent of thioacetic group) was added to the solution to prevent disulfide formation during the reaction. The pH solution was checked, and eventually, NaOH solution was added to maintain the reactant pH solution between 7 and 8. The mixture was left at room temperature and stirred overnight. Afterwards, the solution was saturated with 8 g of sodium chloride, and a liquid–liquid extraction was carried out with 200 mL of dichloromethane. The organic phase was dried with sodium sulphate, filtered, the solvent evaporated, and then precipitated twice in cold diethyl ether. Conversion = 100% (from ^1^HNMR data). Yield = 60%.

^1^H NMR (CDCl_3_): δ = 1.1 (m, 206H, PPG CH_3_ = 69 monomeric units), 3.4 (m, 68H, PPG CH = 68 monomeric units), 3.5 (m, 131H, PPG CH_2_ = 66 monomeric units), 3.65 (m, 924H, PEG CH_2_ = 231 monomeric units), 4.3 (t, 6 H,‒CH_2_CH_2_‒O‒CO‒CH=CH_2_), 5.8 (dd, 6H, CH_2_=CH‒COO‒), 6.15 and 6.4 ppm (both dd, 6H, CH_2_=CH‒COO).

FT-IR (film on ATR plate): 2990–2790 (ν C-H), 1724 (ν C=O), 1467 (δ_s_ CH_2_), 1342, 1279, 1242, 1097 (ν_as_ C-O-C), 962, 841 (ν_s_ C-O-C) cm^−1^.

### 2.4. Synthesis of Pluronic F127 Diacrylate (F127DA)

Pluronic F127DA was synthesized according to a previously reported method [7]. Briefly, 10 g of Pluronic F127 (0.793 mmol, 1.587 meq of –OH groups) was dissolved in 150 mL of toluene under a nitrogen atmosphere and dried by refluxing through a Soxhlet extractor filled with 4 Å molecular sieves. After 3 h, the solution was cooled to room temperature and then immersed in an ice bath. A total of 0.66 mL of triethylamine (4.76 mmol, 2 mol per mol of acryloyl chloride) was added to the reactor while 0.193 mL of acryloyl chloride (2.38 mmol, 1.5 equiv. per –OH group) diluted in 10 mL of toluene was dropped using a dropping funnel. The mixture was stirred overnight, then filtered, and the solvent evaporated at the rotary evaporator. The resulting viscous oil was dissolved in 200 mL of dichloromethane and washed two times with 30 mL of water saturated with sodium chloride. The solution was dried with sodium sulphate, filtered, the solvent evaporated, and then precipitated twice in cold diethyl ether. Conversion = 100% (from ^1^H NMR data). Yield = 80%.

^1^H NMR (CDCl_3_): δ = 1.1 (m, 206H,PPG CH_3_ = 69 monomeric units), 3.4 (m, 68H, PPG CH = 68 monomeric units), 3.5 (m, 131H, PPG CH_2_ = 66 monomeric units), 3.65 (m, 924H, PEG CH_2_ = 131 monomeric units), 4.3(t, 2H,‒CH_2_CH_2_‒O‒CO‒CH=CH_2_), 5.8 (dd, 2H, CH_2_=CH‒COO‒), 6.15 and 6.4 ppm (both dd, 2H, CH_2_=CH‒COO).

FT-IR (film on ATR plate). 2990–2790 (ν C-H), 1724(ν C=O), 1467 (δ_S_ CH2), 1342, 1279, 1242, 1097 (δ_as_ C-O-C), 962, 841 (ν_S_ C-O-C) cm^−1^.

### 2.5. Gel Synthesis by Radical Crosslinking

An appropriate quantity of F127DA was dissolved in an adequate volume of Phosphate-buffered saline (PBS) 10 mM (PH = 7.4) to obtain the desired final concentration (10–30% *w*/*v*) of polymer, and the mixture was left in an ice bath for 30 min until complete dissolution. Then, 0.94 μL of APS and 0.21 μL of TEMED were added to the cold polymer solution, transferred into a 48-well plate (180 μL for each well), and left reacting for 30 min before the gel was collected and used for characterization.

### 2.6. Gel Synthesis by Michael-Type Addition

A total of 0.057 g of F127TA was dispersed in 180 μL (polymer solution concentration: 32% *w*/*v*) of NaOH 0.2 M solution, and the mixture was left in an ice bath (2–3 °C) for 1.5 h under stirring. Meanwhile, 0.024 g (ratio F127HA equivalents/F127TA equivalents = 1) of F127HA was dissolved in 110 μL (polymer solution concentration: 22% *w*/*v*) of PBS 10 mM (pH 7.4). The mixture was left in an ice bath under stirring. The cold polymer solution was buffered with 100 μL of HCl 0.6 M solution and 50 μL of HCl 0.2 M solution to obtain a pH equal to 7.4 (adjusting the pH value with additional drops of the HCl/NaOH solutions when necessary). The final volume of the aqueous solution was adjusted by adding PBS to finally obtain the target polymer concentration of 10–30% *w*/*v*. The two cold solutions, i.e., the activated F127TA and F127HA, were rapidly mixed and stirred. The gelling solution was transferred into an agarose gel mold (Supplementary Materials) or into a 24-well plate (50 μL for each well to obtain a centered drop) and left overnight at 37 °C before characterization.

### 2.7. NMR Diffusion Measurements

The diffusion of ibuprofen in the F127 TA–HA gels was investigated with ^1^H high-resolution magic angle spinning (HR-MAS) NMR spectroscopy. A series of F127 TA–HA gelling solutions with polymer concentrations of 10, 20, and 27% *w*/*v* were prepared according to the procedure described above; all the solutions (NaOH 0.2 M, HCl 0.6 and 0.2 M, and PBS 10 mM) were prepared with D_2_O instead of deionized water. A stock solution of ibuprofen in PBS (300 mg/mL) was prepared and mixed with F127TA and F127HA to obtain a concentration inside the gel of 60 mg/mL (0.27 M). The gelling solutions were left in a thermal bath overnight at physiological temperature (37 °C). After gel formation, a small amount of the gels was used to perform the NMR experiments. The samples were transferred into a 4 mm zirconium rotor containing a volume of approximately 12 μL. All the spectra were recorded on a Bruker Avance DRX spectrometer operating at 500 MHz proton frequency and equipped with a dual ^1^H/^13^C HR-MAS probe head for semisolid samples. The self-diffusion coefficient was measured using pulsed field gradient spin echo (PGSE) techniques (bipolar pulse longitudinal eddy current delay (BPPLED) sequence). In particular, the combination of the high resolution afforded by the HR-MAS set-up with pulse field gradient (PFG) capabilities allows for the diffusion measurements of complex heterogeneous materials. The duration of the magnetic field pulse gradient (Δ) in the MAS direction and the diffusion time (δ) were optimized for each sample in order to obtain complete dephasing of the signals with the maximum gradient strength. The δ values were in the range of 1.7–3 ms, and Δ was 50–200 ms. In each PGSE experiment, a series of 32 spectra with 32 k points were collected. For each experiment, 24 scans were acquired. The pulse gradients were incremented from 2 to 95% of the maximum gradient strength in a linear ramp. The temperature was set at 305° K (32 °C), and the spinning rate was set at 4 kHz. The ^1^H high-resolution PGSE experiments on ibuprofen dissolved in D_2_O solution were performed using a high-resolution 5 mm broadband inverse probe head. Experimental conditions similar to those described for the gel samples were also used for the solution sample.

### 2.8. Gel Point Characterization

The crosslinking kinetics and gel point were studied through microrheology via dynamic light scattering analysis (Malvern Zetasizer Nano ZS). Monodisperse gold nanoparticles (Au NPs, 40 nm diameter, stabilized suspension in 0.1 mM PBS, reactant free, Sigma) and poly(ethylene glycol)methyl ether thiol (MW = 2000) were purchased from Sigma-Aldrich and used as received. A total of 2 mL of Au NPs was mixed with 54 μg of poly(ethylene glycol)methyl ether thiol (ligand concentration 0.0135 μmol/NPs mL). The solution was stirred at room temperature for two hours and purified with dialysis against deionized water for 24 h (dialysis membrane: Cellu-Sep T1/Nominal MWCO: 3500). After the purification, the PEGylated Au NPs were transferred into vials and stored in a refrigerator. Gelation of the F127DA and F127 TA-HA solutions at target polymer concentrations was obtained as described above. In order to encapsulate the PEGylated Au NPs and to obtain a final gel volume of 1 mL in the DLS cuvette, the curing buffer was premixed with 500 μL of the 10 nm PEGylated Au NPs suspension. The gel crosslinking kinetics were studied by evaluating the increase in the viscosity of the system by measuring via DLS the the diffusion coefficient *D* of the Au NPs (fixed radius, *R_h_* = 20 nm) dispersed in the reacting solution and applying the Stokes–Einstein equation $\eta =\frac{K_{B}T}{6\pi R_{h}D}$ to determine the viscosity *η* (*K_B_* is the Boltzmann constant, and T is the temperature).

### 2.9. Swelling Measurements

A series of F127 DA and TA–HA gelling solutions were prepared according to the procedure described above with different polymer concentrations (10–30% *w*/*v*). Each gelling solution was transferred into the molds obtained by shaping 140 μL of cylindrical wells made of agarose gel, diameter 1.2 cm (Supplementary Materials). The mold was left overnight in an oven at 37 °C to allow the complete hydrogel formation. Then, each gel was extracted from the mold, and their weight was measured with a high-precision balance. Afterwards, the gels were transferred into a 6-well plate and filled with 5 mL of PBS 10 mM solution (pH 7.4). The weight measurements of the swollen gels were carried out after 24 h with the expectation that this time was sufficient to achieve an equilibrium condition. The weight measurements of the gels were always carried out after removing the excess water, which was often present around the gel surface, with blotting paper. The swelling degree was defined as $\%\;degree\;of\;swelling=\frac{\Delta w}{w_{0}}\cdot 100$, where $w_{0}$ is the initial gel weight, and $\Delta w=w-w_{0}$; it was measured at both room temperature (25 °C) and physiological temperature (37 °C). The degree of swelling on the basis of the dry polymer was also calculated as follows: $Sw\left(\%\right)=\frac{\Delta w}{w_{0,dry}}\cdot 100$, where $w_{0,dry}$ is the dry mass of the polymer, and $\Delta w=w-w_{0,dry}$; the data are shown in Figure S5, Supplementary Materials.

Three different samples were tested at each temperature to obtain the mean values and standard deviations.

### 2.10. Cell Adhesion Tests

A total of 50 μL of the Michael-type (F127 TA–HA) or radical (F127DA) gelling solution (10% *w*/*v*) was transferred into a 24-well plate to generate a gel spot at the center of each well. The plate was incubated at 37 °C overnight, and the gel was washed six times every 10 min with 500 μL PBS 10 mM. When required, after 10 min, the gelling drops were pretreated and left overnight with either 500 μL of cell medium or RGD peptide (GRGDSPC [22], Caslo ApS, DK) solution in PBS (20 mg/mL). Afterwards, 16,000 cells (3T3 fibroblast cell line (ATCC number: CRL-1658), or EOMA endothelial cell line (ATCC number: CRL-2586), purchased form LGC Standard S.r.l. (Sesto San Giovanni, Milan, Italy),were seeded into each well and incubated at 37 °C in culture medium (DMEM supplemented with 10% *v*/*v* fetal bovine serum). After 24–96 h, gel morphology and cell spreading were assessed via optical microscopy. Images were acquired with a Zeiss AxioObserver microscope equipped with a high-resolution digital videocamera (AxioCam, Zeiss).

## 3. Results and Discussion

### 3.1. Synthesis of the Polymeric Precursors

**Figure 1:**
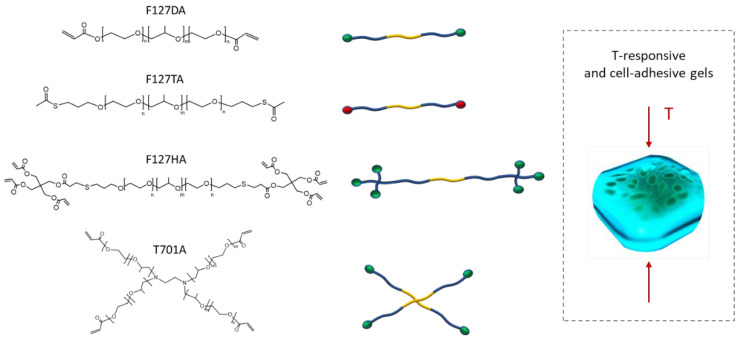
Structures of the synthesized F127-based gel precursors used to obtain thermoresponsive and cell-adhesive gels.

Different Pluronic precursors were designed and synthesized to obtain hydrogels through either Michael-type addition or radical crosslinking (Figure 1). F127 diacrylate (F127DA) was synthesized via esterification by functionalization of the Pluronic terminal hydroxyl groups with acrylates through a direct reaction with acryloyl chloride [7]. This polymer was further used for the radical crosslinking method. In order to obtain precursors that could crosslink via Michael-type addition, it was necessary to include more functional sites in each polymer chain, such as acrylates and thiols [7,11]. In this study, we designed a linear F127 precursor that contained two terminal thiol groups and a macromolecular multifunctional Michael acceptor. The Pluronic dithiol was obtained from F127 thioacetate (F127TA), which has the advantage of being a protected and stable derivative that can be converted in situ into F127 dithiol by treatment with a base [11]. On the other hand, a Pluronic crosslinker, which presents six acrylates per chain, was designed (F127HA). This precursor was synthesized from F127TA, which was deprotected with 0.2 M NaOH, and reacted via Michael-type addition with a large excess of commercial tetraacrylate (pentaerythritol tetraacrylate) to obtain a Pluronic with three acrylates per terminus. An alternative approach was also investigated that involved the synthesis of a Tetronic tetra acrylate (T701A). This precursor was obtained by reaction of the four terminal hydroxyl groups of the commercially available Tetronic T701 with acryloyl chloride under the same conditions tested for the synthesis of F127DA (Supplementary Materials).

**Figure 2:**
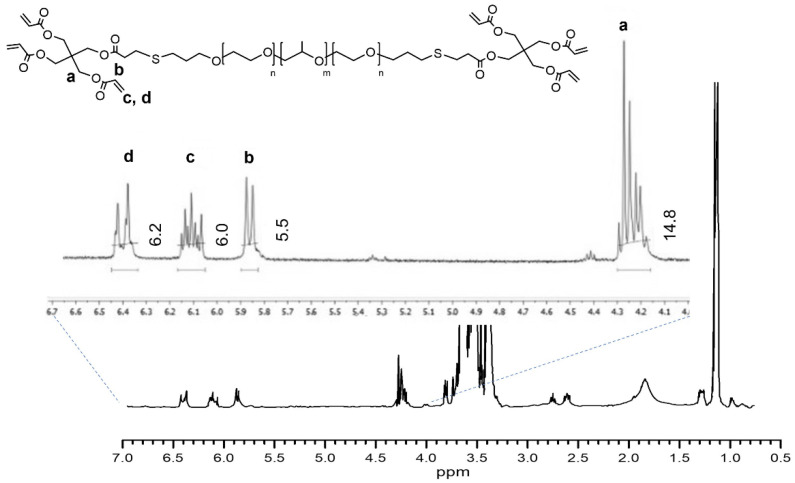
^1^H NMR (CDCl3) of F127HA with enlargement of the chemical shifts corresponding to the acrylate groups.

The characterization of the precursor F127HA required an accurate analysis. ^1^H NMR confirmed the presence of pentaerythritol tetraacrylate conjugated to the F127 termini (Figure 2), while size exclusion chromatography (SEC) was used to characterize the final molecular weight and dispersity.

**Figure 3:**
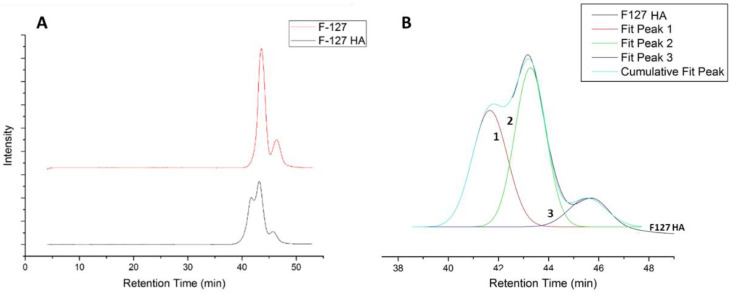
(**A**) F127HA (black line) and F127 (red) SEC chromatograms. (**B**) Peak deconvolution of F127HA chromatogram. (**C**) Structure of the dimeric F127-based macromonomer corresponding to the highest molecular weight population.

In fact, the addition of pentaerythritol tetraacrylate to multiple chains of F127 thiol may result in the formation of larger macromolecules despite the use of a significant excess of this reagent to minimize such side products. The SEC analysis reveals the existence of a population with a molecular weight greater than that of a single F127 chain, despite its relatively low dispersity (Figure 3 and Table 1).

By deconvoluting the SEC chromatograms, it was possible to determine the molecular weight distribution of three distinct populations, two of which belonged to the commercial F127 starting material with the expected hexa-acylated pattern. Notably, the highest molecular weight peak suggested the potential existence of a dimeric macromolecule, as proposed in Figure 3C.

However, the presence of this larger polymer specie in the precursor did not affect the final crosslinking of the gel, and F127HA was further used as obtained.

### 3.2. Deprotection of Pluronic Thioacetate

**Figure 4:**
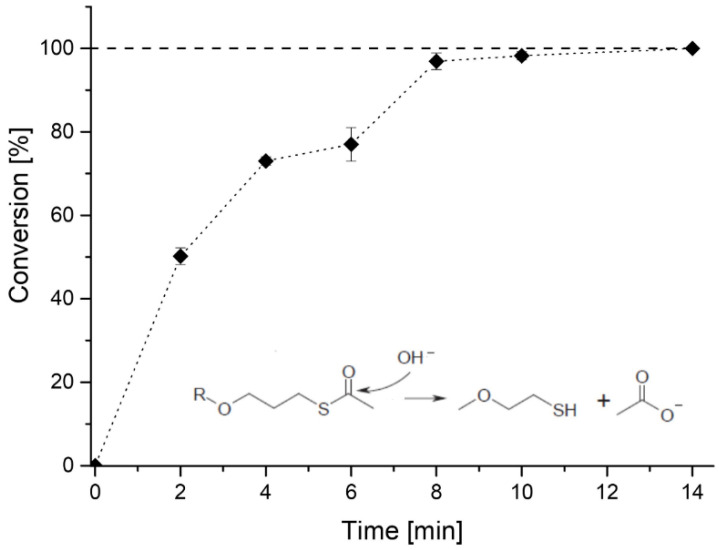
F127TA 20% *w*/*v* deprotection kinetics in 0.2 M NaOH solution in D_2_O.

The hydrolysis of thioacetate groups was essential to obtain an efficient chemical gelation, and this can be easily accomplished by the use of a strong base such as NaOH [18]. The primary goal was to achieve complete deprotection in the shortest reaction time to prevent the formation of disulfides, which would affect the efficiency of the Michael-type addition during gel formation. This hydrolytic process depends on three main variables, i.e., the concentration of the base, the concentration of the protected macromolecule, and the reaction time. The kinetic study was conducted for a solution concentration of F127TA equal to 20% *w*/*v*. The NaOH solution was prepared with D_2_O to quantify thioacetate hydrolysis via ^1^H-NMR. NaOH 0.2 M was used since, at this concentration, the base was relatively easy to neutralize after deprotection. This hydrolysis follows a pseudo first-order kinetics with respect to the concentration of both thioacetate (and thus polymer) and the base (Figure S1, Supplementary Materials). Under these reaction conditions, complete deprotection was achieved in ~10 min, and this was the time used for activating the F127TA precursor during gel formation (Figure 4).

### 3.3. Gel Preparation

F127-based hydrogels were obtained via Michael-type addition by mixing a solution of deprotected F127TA to F127HA (same equivalents of thiol and acrylate groups) at a tunable concentration of 10–30% *w*/*v*, buffering in water at physiological pH via PBS 10 mM (pH 7.4), and, finally, leaving overnight at 37 °C to allow complete hydrogel formation. We also investigated the feasibility of obtaining Michael-type gels by using T701A as a four-arm crosslinker rather than F127HA. A hydrogel was obtained, although its physicochemical characteristics were not acceptable for any further investigation or application. In fact, the final gel was opaque, sticky, and soft (Figure S2, Supplementary Materials). The poor quality of the material may be ascribed to the low solubility in water of this compound, which may lead to partial phase separation during crosslinking.

Alternatively, F127 gels were also obtained by radical crosslinking of F127DA (polymer concentration 10–30% *w*/*v*). In this case, a standard initiator/co-initiator system based on the APS/TEMED pair was used [23,24].

### 3.4. Crosslinking Kinetics

The crosslinking kinetics were studied with dynamic light scattering through a microrheological approach [25] by evaluating the increase in the viscosity of the system until the gel network was obtained. This was achieved by measuring the diffusion coefficient of the monodispersed PEGylated gold nanoparticles suspended in the reacting polymer solution and using the Stokes–Einstein equation to evaluate the viscosity of the system [26].

**Figure 5:**
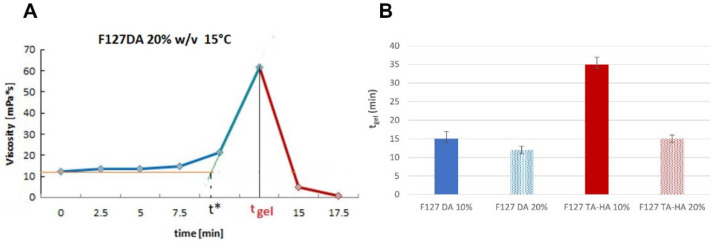
(**A**) Viscosity values of the F12DA gel precursor during crosslinking. (**B**) Calculated t_gel_ from different F127 precursors reacting at 15 °C.

As the sol–gel transition occurred during crosslinking, the viscosity values rapidly increased (time point t*, Figure 5A) until the autocorrelation function was unable to characterize the scattering of the Au particles independently from the gel network (Figure S3, Supplementary Materials), and the apparent viscosity values decayed. This transition was used to identify the gel point (t_gel_).

The tests on different gelling precursors were carried out under conditions where the fast physical (thermal) gelation was avoided during chemical crosslinking (15 °C, polymer concentration 10–20% *w*/*v*). The radical crosslinking of F127DA at 10% *w*/*v* showed a gel point at approximately 15 min, which decreased to 12 min when the polymer concentration was 20% *w*/*v* (Figure 5B). Michael-type gelation was slower (35 min) for the F127 TA–HA precursors at 10% *w*/*v*, which became more rapid (gel point at 15 min) when the polymer concentration was increased to 20%.

### 3.5. Swelling Tests

**Figure 6:**
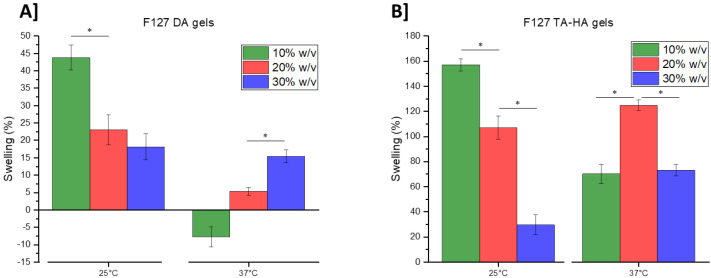
Degree of swelling (%) of (**A**) F127DA gels and (**B**) F127 TA–HT gels (10–30% *w*/*v*) at room temperature (25 °C) and at physiological temperature (37 °C). * *p* < 0.05, using Student’s *t*-test. Error bars shown are SD.

The degree of swelling of the F127 gels, obtained by radical crosslinking and by Michael-type addition, was measured at different polymer concentrations and temperatures, and the results are reported in Figure 6.

It was observed that the swelling degree of the F127DA gels decreased with increasing concentration due to the rise of physical crosslinking, as proven by the dependence of the physical gel transition temperature (T_1_) [27] to the concentration of F127 in water [7,11]. This was particularly evident for F127 concentrations ≥20% *w*/*v* and ≥25 °C. Interestingly, the 10% *w*/*v* sample showed a shrinkage effect at 37 °C due to the temperature transition. This shrinkage was less pronounced for higher polymer concentrations, which was likely due to a concurrent increase in osmotic pressure that tended to increase swelling. Regarding the F127 TA–HA gels, the degree of swelling at room temperature (25 °C) increased as the Pluronic concentration decreased, which was an effect of the chemical crosslinking density and the dependence of physical crosslinking on the F127 concentration. The swelling of these gels was more pronounced than that of the F127DA gels at the same concentration and temperature, and this could be ascribed to a lower crosslinking density due to the different crosslinking method used. At physiological temperature (37 °C), the lower critical solution temperature (LCST) of F127 [28,29] led to an increase in hydrophobic interactions between polymer chains, causing the swelling degree of the F127 TA–HA gels at 10% *w*/*v* to decrease. However, the 20% *w*/*v* and 30% *w*/*v* gels did not exhibit this behavior. In fact, the swelling degree of the 30% *w*/*v* gels almost doubled from room to physiological temperature. This inverse trend could be ascribed to an effect of hydrolysis (due to the incubation at 37 °C during gel preparation) of the ester bonds of the crosslinks, which are unstable at physiological temperature because of the neighboring sulphides generated by the Michael-type addition [30]. Clearly, this hydrolysis may be favored by the higher concentrations of esters in the gels, and therefore, the effect of a reduction in crosslinking density would be more significant for the 30% *w*/*v* samples.

### 3.6. Diffusion Measurements

**Figure 7:**
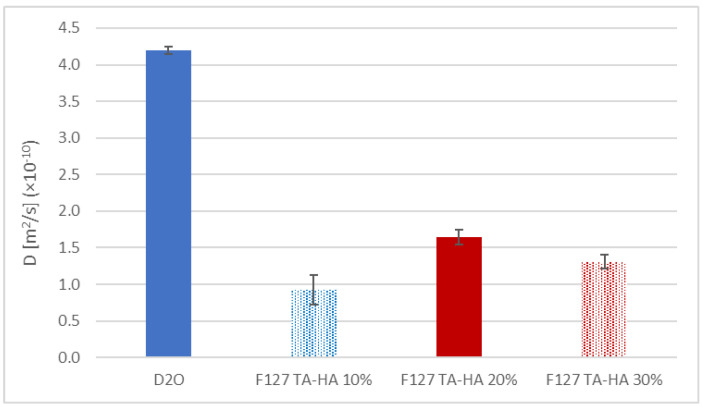
Diffusion coefficients for Ibuprofen in D_2_O and in the F127 TA–HA gels (10–30% *w*/*v*) obtained with HR-MAS NMR spectroscopy.

In order to assess the impact of hydrolysis on the gel network of the Michael-type gels, specifically in terms of drug release properties, we analyzed the diffusion behavior of a standard low molecular weight drug (Ibuprofen) loaded in the gel phase using 1H HR-MAS PGSE NMR spectroscopy [31,32]. This behavior was compared with the free diffusion of the drug in a water solution. The diffusion coefficient (D) of ibuprofen obtained in deuterated water (D_2_O) and in the F127 TA–HA gels prepared at different polymer concentrations is reported in Figure 7.

The results confirmed the trend observed in the swelling tests, since the gel at 10% *w/v* showed the lowest diffusion coefficient because of a relatively small pore size. The gel at 20% provided the highest diffusion coefficient, indicating an increase in porosity due its partial degradation during the incubation at 37 °C, an effect which was less pronounced with the gel at 30%. Clearly, in all cases, the diffusion coefficient of the drug inside the gel network due to drug-confinement or drug-polymer interactions was lower than the value measured in the pure D_2_O solution.

### 3.7. Cell Adhesion Tests

For applications in tissue engineering, cell encapsulation, and as material for cell culture, Pluronic gels should provide cell adhesion [33,34]. Therefore, we assessed this property by pre-forming gels in 24-well culture plates before seeding and incubating the cells. First, the F127DA gels obtained via radical crosslinking were tested; Pluronic gels formed without any pretreatment were compared with Pluronic gels crosslinked in the presence of cell culture medium containing fetal bovine serum (FBS), and their effects were investigated on 3T3 fibroblasts [35] and EOMA endothelial cell cultures [36].

**Figure 8:**
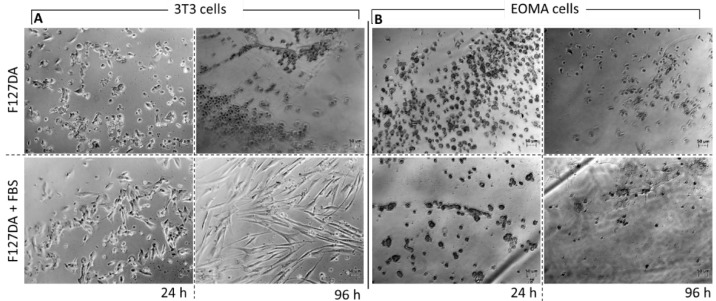
3T3 Fibroblasts (**A**) and EOMA cells (**B**) cultured on F127 DA gels (10% *w*/*v*) and F127 DA gels pretreated with FBS culture medium. Cells were incubated and evaluated at 24 h and 96 h (right). Scale bar 50 μm.

The non-pretreated gels did not show any adhesion effect, resulting in a round cell shape that indicated no adhesion and, most likely, cell death (Figure 8). This result confirmed the non-adhesive property of Pluronic (i.e., PEG-based) gels when no additional adhesion signals are introduced. On the other hand, the FBS-pretreated gels induced cell spreading, particularly after 96 h, as the fibroblasts presented their typical elongated shape (Figure 8A). This suggested that the serum proteins premixed during the crosslinking reaction may have been embedded in the final gel and were able to promote cell adhesion due to their integrin binding domains.

The EOMA cells showed a similar behavior with no adhesion on the F127DA gels without pretreatment and negligible adhesion at 96 h on the gels pretreated with FBS (Figure 8B). This low performance on endothelial cells was probably due to the lower adaption capabilities of endothelium cells to adhere on these gels compared to those of fibroblasts, considering the relatively simple adhesion signaling used.

Second, we assessed the adhesion properties of the Michael-type crosslinked gels. The Pluronic TA–HA gels that were formed without any pretreatment were compared with the gels crosslinked in the presence of cell culture medium containing FBS. In this case, the Michael-type addition reaction offered the possibility to introduce cysteine-containing peptides to conjugate cell-active moieties to the gel network during crosslinking. Therefore, we crosslinked the gels in the presence of cysteine-terminated RGD peptide (GRGDSPC) as a common recognition motif found in several extracellular matrix proteins. The characteristics of these gels were investigated on 3T3 fibroblasts and EOMA endothelial cell cultures.

**Figure 9:**
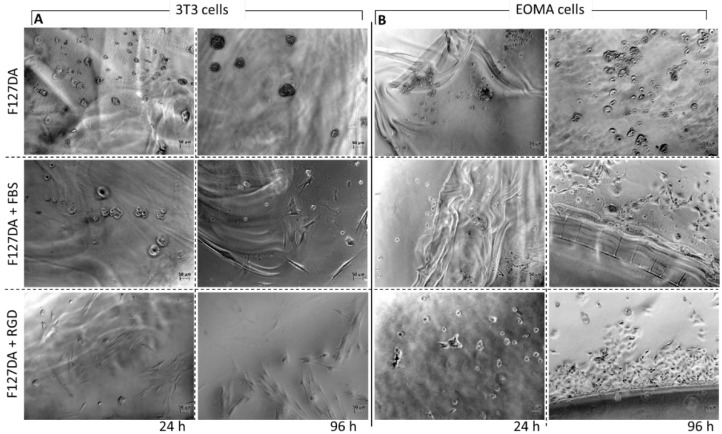
3T3 Fibroblasts (**A**) and EOMA cells (**B**) cultured on F127 TA–HA gels (10% *w*/*v*), F127 TA–HA gels pretreated with FBS culture medium, and F127 TA–HA gels pretreated with RGD peptide. Cells were incubated and evaluated at 24 h and 96 h (right). Scale bar 50 μm.

Similarly, as with the radical-crosslinked gels, the non-pretreated Michael-type gels did not show any adhesion effect on the fibroblasts, resulting in a round cell shape, while the medium pretreated gels induced cell spreading after 96 h (Figure 9A).

As expected, RGD peptide conjugation enhanced the adhesion properties of the gels, showing a clear effect already at 24 h, which improved after 96 h (Figure 9A).

Endothelial cells showed a similar behavior with no adhesion on the F127 TA–HA gels and cell spreading at 96 h on the gel pretreated with medium (Figure 9B). With RGD peptide, cell spreading was partially evident only at 96 h, indicating a less pronounced cell–gel interaction with this cell type, thus confirming the lower adaption capabilities of endothelium cells to adhere on these Pluronic gels compared to those of fibroblasts, taking into account the simple integrin-binding domains provided.

## 4. Conclusions

Functional Pluronic F127 precursors were successfully designed and synthesized for the preparation of Pluronic-based thermoresponsive hydrogels. Linear F127 thioacetate and F127 multi-acrylate were used to achieve F127-based hydrogels through Michael-type addition. The macromolecular architecture of the precursors was optimized to obtain good gelation kinetics, evidence of thermal response on swelling, and drug diffusion within the gel network for release. The properties of these gels were compared to those created through free radical crosslinking of F127 diacrylate. Chemical gelation took place within 15–35 min, depending on the crosslinking method. Gel swelling was affected by temperature variations between 25 and 37 °C and by possible partial degradation of Michael-type crosslinks at physiological temperature, as confirmed with Ibuprofen diffusion tests via HR-MAS NMR. In vitro tests were conducted on fibroblasts and endothelial cells to evaluate the gel response to cellular adhesion. By using gels pretreated with serum proteins or functionalized with an RGD peptide, cell adhesion and spreading were clearly promoted. This study offers a method for creating customized hydrogels suitable for a range of biomedical applications, including soft-tissue engineering, cell encapsulation, wound healing, and sustained delivery of therapeutic molecules.

## Figures and Tables

**Table 1:** Number average, weight average molecular weight, and dispersity of F127 and F127HA with analysis of each deconvolute peak form SEC chromatogram.

	F127	Peak 1	Peak 2	F127HA	Peak 1	Peak 2	Peak 3
$\bar{M_{n}}$	13,200	16,500	7100	20,000	29,700	18,200	8900
$\bar{M_{w}}$	15,500	17,200	7500	23,600	31,300	19,000	9600
Ð	1.17	1.04	1.06	1.19	1.05	1.04	1.08

## Data Availability

The data presented in this study are available on request from the corresponding author.

## Supplementary Materials

The following supporting information can be downloaded at: https://www.mdpi.com/article/10.3390/ma16072749/s1. Figure S1. Kinetic plot of F127TA hydrolysis. 20% *w*/*v* of 127TA was dissolved in 0.2 M NaOH in D_2_O, and the concentration of tioacetate (C) was monitored over time by 1H NMR. Figure S2. (A) hydrogel obtained from F127TA and T701A as polymer precursors. (B) hydrogel obtained from F127 DA as polymer precursor. Figure S3. Autocorrelation function recorded by DLS from F127DA 10% *w*/*v* reacting solution at 15 °C, before gel point (10 min) and after gel point (20 min). Figure S4. (A) typical process scheme for F127-based gel preparation. (B) agarose gel mould (cylindrical hole diameter 1.2 cm) used for shaping F127-based hydrogels. Figure S5. Degree of swelling (%) of (A) F127DA gels and (B) F127 TA-HT gels (10–30% *w*/*v*) at room temperature (25 °C) and at physiological temperature (37 °C). The degree of swelling *Sw* was calculated as follows: $Sw\left(\%\right)=\frac{\Delta w}{w_{0,dry}}\cdot 100$, where $w_{0,dry}$ is the dry mass of the polymer and $\Delta w=w-w_{0,dry}$, where *w* is the mass of the swollen gel. Table S1. Average diffusion coefficients (D) for Ibuprofen in D2O and in F127 TA-HA gels (10–30% *w*/*v*), obtained by HR MAS NMR spectroscopy (SD = standard deviation).
