# Supplementary material for: Design of Functional Pluronic-Based Precursors for Tailoring Hydrogel Thermoresponsiveness and Cell-Adhesive Properties
Source: Materials (Basel). 2023 Mar 29;16(7):2749. doi: 10.3390/ma16072749 (PMC10095789; doi:10.3390/ma16072749)
Supplement: Supplementary file 1 [file materials-16-02749-s001.zip › materials-2282206-SI.pdf]

Supporting Information

Design of functional Pluronic-based precursors for tailoring hydrogel thermoresponsiveness and cell-adhesive properties.

Giulia Camana[a#], Mirko Tavano[a#], Min Li[b], Franca Castiglione[a], Filippo Rossi[a], Francesco Cellesi[a*]

[a]*Dipartimento di Chimica, Materiali ed Ingegneria Chimica "G. Natta", Politecnico di Milano, Via Mancinelli 7, 20131 Milan, Italy;*
[b]*Renal Research Laboratory, Fondazione IRCCS Ca' Granda Ospedale Maggiore Policlinico, Via Pace 9, 20122 Milan, Italy;*

[#]equally contributed.

[*]Correspondence: francesco.cellesi@polimi.it

Synthesis of Tetronic 701 acrylate (T701ACR)

10 g of T701 (2.78 mmol, 11.1 meq –OH groups) were dissolved in 150 mL of toluene under nitrogen atmosphere and dried refluxing toluene through a Soxhlet extractor filled with 4 Å molecular sieves. After 3 hours the solution was cooled at room temperature and then immersed in an ice bath. 5.3 mL (38.2 mmol, 1.5 mol per mol acryloyl chloride) of triethylamine was added into the reactor, 2 mL of acryloyl chloride (25.5 mmol, 2.3 eq per –OH group) diluted in 10 mL of toluene were dropped using a dropping funnel. The dropping funnel was washed with additional 10 mL of toluene, which were dropped into the reactor under nitrogen flux, and the mixture was stirred overnight at room temperature.

The mixture was filtered using Buchner filter and the solvent evaporated at the rotary evaporator. The resulting viscous oil was dissolved in 200 mL of DCM and washed two times with 30 mL of deionized water saturated with sodium chloride. The solution was dried with sodium sulphate, filtered, the solvent evaporated and then precipitated two times in cold diethyl ether. Conversion = 100 % (from ^{1}HNMR data). Yield = 80%.

^{1}H NMR ($CDCl_3$): δ=1.1 (m, 159H, PPG C**H_3** = 53 monomeric units), 3.4 (m, 50H, PPG C**H** = 50 monomeric units), 3.5 (m, 89 H, PPG C**H_2** = 45 monomeric units), 3.65 (m, 40H, PEG C**H_2** = 10 monomeric units), 4.3 (t, 4H,–CH_2C**H_2**–O–CO–CH=CH_2), 5.8 (dd, 4H, CH_2=C**H**–COO–), 6.15 and 6.4 ppm (both dd, 4H, C**H_2**=CH–COO).

FT-IR (film on ATR plate): 2990-2790 (ν C-H), 1467 (δ_s CH_2), 1724 (ν C=O), 1342, 1279, 1242, 1097 (ν_{as} C-O-C), 962, 841 (ν_s C-O-C) cm^{-1}.

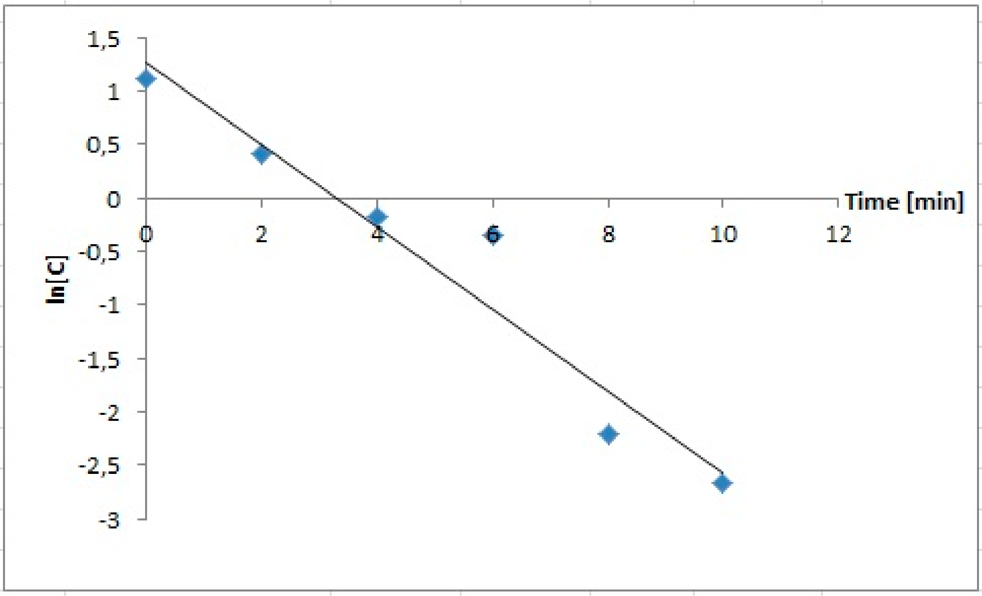

Figure S1. Kinetic plot of F127TA hydrolysis. 20% w/v of 127TA was dissolved in 0.2M NaOH in D_2O, and the concentration of tioacetate (C) was monitored over time by ^{1}H NMR.

A **B**

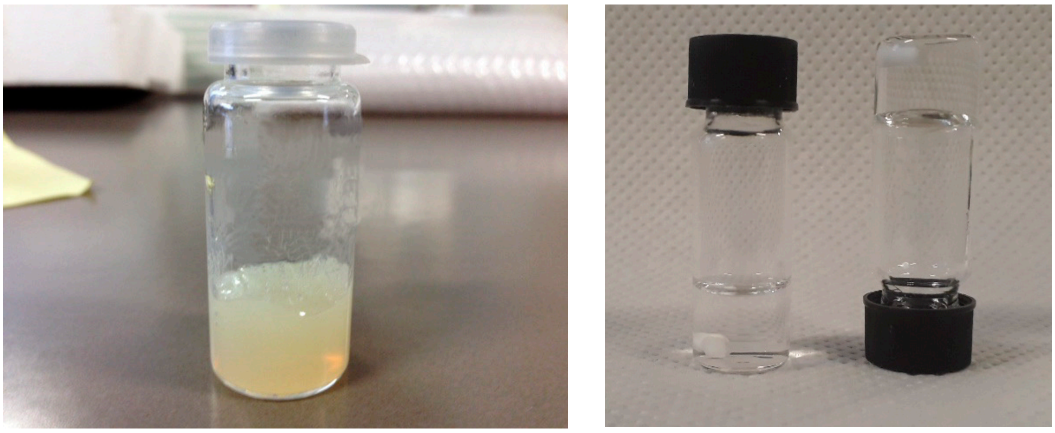

Figure S2. A) hydrogel obtained from F127TA and T701A as polymer precursors. B) hydrogel obtained from F127 DA as polymer precursor.

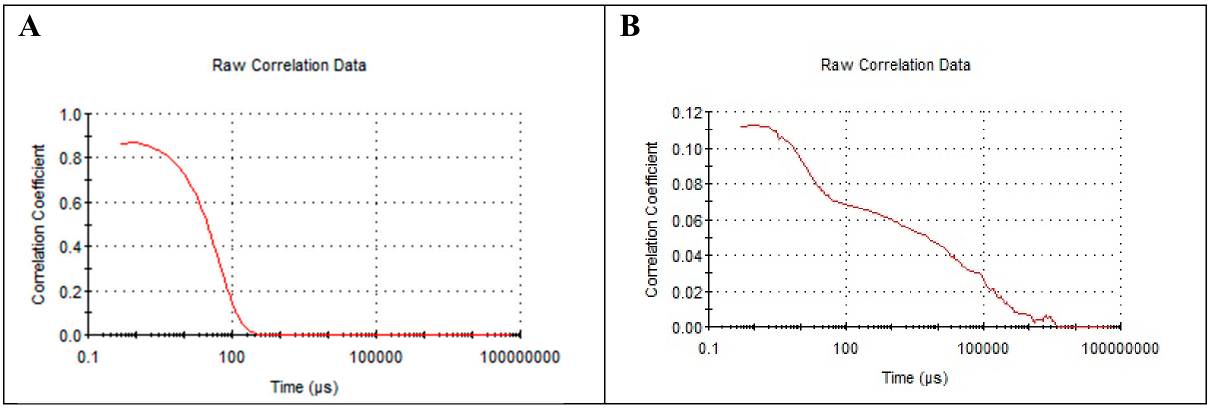

Figure S3. Autocorrelation function recorded by DLS from F127DA 10% w/v reacting solution at 15°C, before gel point (10 min) and after gel point (20 min).

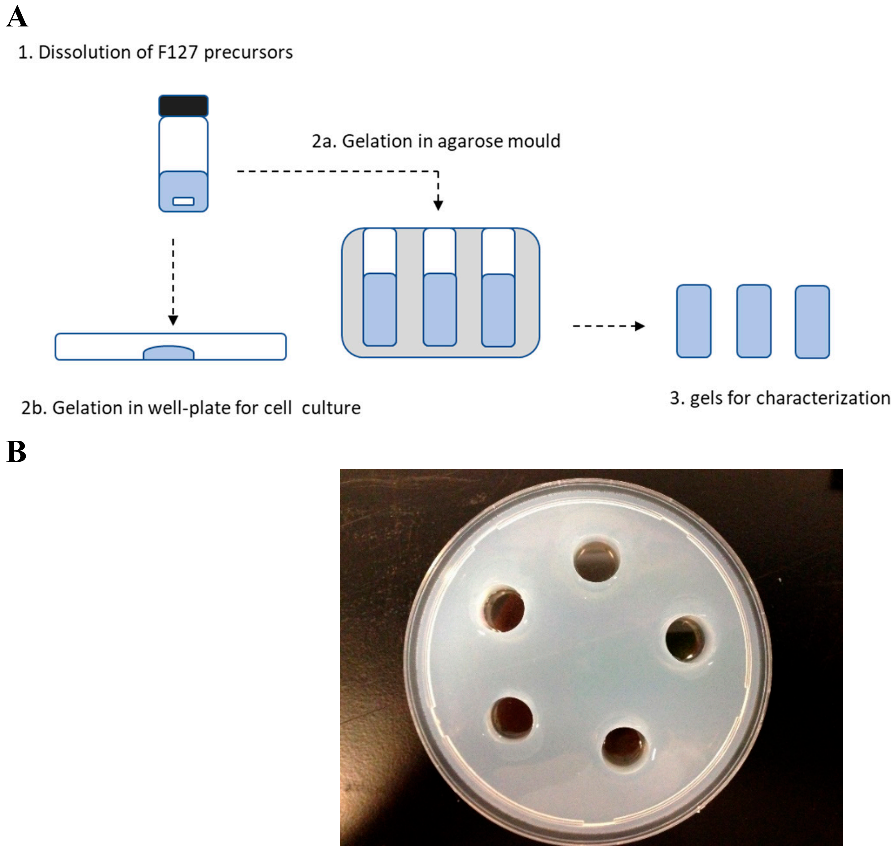

Figure S4. A) typical process scheme for F127-based gel preparation. B) agarose gel mould (cylindrical hole diameter 1.2 cm) used for shaping F127-based hydrogels.

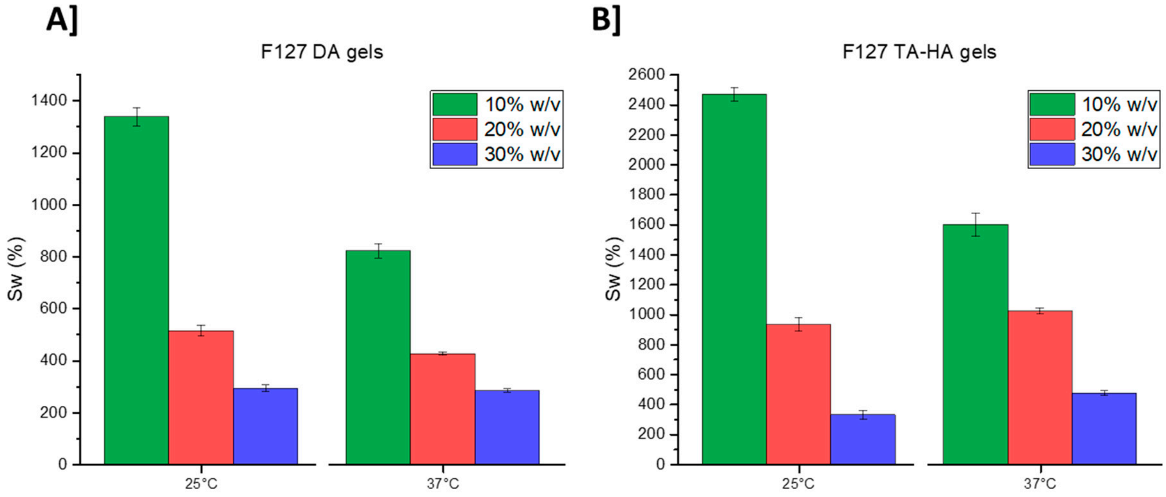

Figure S5. Degree of swelling (%) of A) F127DA gels and B) F127 TA-HT gels (10-30% w/v) at room temperature (25°C) and at physiological temperature (37°C). The degree of swelling *Sw* was calculated as follows: $Sw(\%) = \frac{\Delta w}{w_{0,dry}} \cdot 100$, where $w_{0,dry}$ is the dry mass of the polymer and $\Delta w = w - w_{0,dry}$, where *w* is the mass of the swollen gel.

Table S1. Average diffusion coefficients (D) for Ibuprofen in D2O and in F127 TA-HA gels (10-30% w/v), obtained by HR MAS NMR spectroscopy (SD = standard deviation).

	D [m^2/s](*10^{-10})	SD
D_2O	4.20	0.05
F127 TA-HA 10%	0.93	0.2
F127 TA-HA 20%	1.65	0.1
F127 TA-HA 30%	1.31	0.1
